# Individualized Care in Nursing Homes Before and After the COVID-19 Pandemic

**DOI:** 10.3390/nursrep14040283

**Published:** 2024-12-06

**Authors:** Aurora García-Camacha Gutiérrez, Irene García-Camacha Gutiérrez, Riitta Suhonen, Beatriz Rodríguez-Martín

**Affiliations:** 1 Social and Health Research Center, University of Castilla-La Mancha, 16002 Cuenca, Spain; aurora.garciacamacha@alu.uclm.es; 2 Department of Mathematics, University of Castilla-La Mancha, 13005 Ciudad Real, Spain; 3 Department of Nursing Science, University of Turku, 20014 Turku, Finland; riisuh@utu.fi; 4 Turku University Hospital, The Wellbeing Services County of Southwest Finland, 20014 Turku, Finland; 5 Department of Nursing and Physiotherapy, Faculty of Health Sciences, University of Castilla-La Mancha, 45600 Talavera de la Reina, Spain; beatriz.rmartin@uclm.es; 6 Research Network on Chronicity, Primary Care, and Health Promotion (RICAPPS), 48902 Barakaldo, Spain; 7 ABC-Age Research Group, University of Castilla-La Mancha, 16002 Cuenca, Spain

**Keywords:** elderly individuals, individualized care, quality of life, environment, COVID-19, nursing care

## Abstract

**Background**: Individualizing care is the essence of nursing, and its benefits have been extensively proven in older people. The changes arisen during the COVID-19 pandemic may have affected it. The aim of this study is to analyze the changes produced in the perceptions about the individualization of care, quality of life, and care environment of elderly people living in long-term care centers before and after the pandemic. **Methods**: A prospective cross-sectional observational study was carried out. For data collection, the Individualized Care Scale-patient, the EuroQol-5D scale, and a reduced version of the Sheffield Care Environment Assessment Matrix test were used, and a statistical analysis was performed. **Results**: A total of 177 people participated in the study, with 87 pre-COVID-19 and 90 post-COVID-19, 62.7% of whom were women. The average age was 83.3 years. General activities of the individualized care obtained medians of 4, 2.5, and 3 (out of 5) in clinical situation, personal life situation, and decisional control dimensions, respectively, and no substantial change was observed pre- and post-pandemic. Nevertheless, 10 out of 17 items related with the maintenance of individuality in the last shift were higher rated after COVID-19. They are mainly related to the feelings and needs of care, daily life activities, and the expression of opinions. The median of all items was 3 despite the improvement observed after the pandemic. Residents scored an average of 6.47 points (out of 10) in the life quality self-evaluation and were satisfied with the care environment (94%). Patients with higher life quality and adherence to their environment perceived better care. **Conclusions**: Although slight improvements were observed in the individualized care after the pandemic, the obtained results revealed that there is still room for improvement. Particularly, it is necessary to develop strategies aimed at motivating the family participation or providing individual spaces in the residences.

## 1. Introduction

Population aging is a phenomenon of increasing magnitude in almost all countries of the world [1]. Between 2020 and 2050, the proportion of the world’s population aged 65 or over is expected to increase from 9.3% to 16.0% [2]. Among the main causes of population aging are the increase in life expectancy and the aging of people born in the “baby boom” after the Second World War [3]. Life expectancy in Spain was 83.03 years in 2022 [4], and it is estimated that by 2040, Spain will surpass countries such as Japan or Switzerland in longevity [5]. According to different studies, the increase in life expectancy is linked to the increase in disability and dependence of older people [6], making it necessary to develop appropriate care plans for the upcoming challenge [7].

In March 2020, the World Health Organization (WHO) declared the COVID-19 pandemic [3], which particularly affected older people and specifically institutionalized people, increasing the mortality rate in this population group [8,9]. At the beginning of the pandemic, most of the global population was forced to remain confined to their homes, with residents of long-term care centers being one of the most affected groups due to the stricter measures they suffered, such as maintaining longer isolation [10,11]. From 2023, the situation normalized [12], and despite the high mortality caused by the pandemic in older people, the Spanish population continued to be among the twenty-five countries with the oldest population [13].

Before the pandemic, attention was dominated by purely clinical tasks and procedures [14], but currently, new strategies are being sought in long-term care centers for the older people to adapt and adjust care to the individual characteristics of users and improve their quality of life [15]. Individualized care considers the personal needs of patients, their clinical conditions, their personal histories, and their preferences, thus promoting their participation in the decision-making process [16]. However, in the face of population aging, governments are encouraging initiatives aimed at promoting healthy aging, such as person-centered care, in which individualized care plays an essential role [17]. The recent pandemic underscores the need for governments to develop programs that address aspects that have worsened with COVID-19, such as isolation and loneliness in older people, while promoting positive changes, such as the incorporation of technologies to reach this population group [18].

It is known that the individualization of care is a fundamental principle in nursing, having reported significant benefits for patients by improving their empowerment and autonomy. Individualized care has been well studied from the perspective of professionals, so it is thought that providing individualized care improves job satisfaction and reduces stress in nursing professionals [19]. Furthermore, various studies highlight the importance of analyzing the provision of individualized care from the patient’s perspective [20]. It is also known that person-centered care is positively associated with the ability to self-care [21] and satisfaction with the care received in older people [22]. In addition, individualized care improves patients’ adherence to the care plan [23], reducing the use of medical services and hospital readmissions [24]. Despite the existing evidence, it is necessary to continue deepening the analysis of individualized care in older people, especially institutionalized ones, as they may have more complex care needs than other population groups [25].

In relation to the instruments used for studies on individualized care from the patient’s perspective, the Individualized Care Scale-patient version (ICS-patient) stands out, which is a validated instrument to evaluate patients’ perceptions of individualized care [26]. This instrument was initially developed in Finnish and is currently validated in several languages, including Spanish [27]. It is useful for understanding and improving the areas where patients’ score for individualized care was worse and knowing the factors that influence them, key information that will contribute to improving the quality of care, and ensuring the patient’s right to be respected as an individual, especially in areas of care, such as long-term care for the elderly.

On the other hand, the importance of the environment where care is provided is known. Accordingly, having an adequate environment in long-term care centers for the older people promotes individualized care, facilitating self-management and independence in the person. Among the scales to measure the care environment, the Sheffield Care Environment Assessment Matrix (A-SCEAM) stands out for its ability to describe the current state of environmental quality and the organization of the center [28].

Improving the quality of care requires that nursing professionals know the factors that influence patient satisfaction. Quality of life is one of the elements beneficially related to achieving quality individualized care, which has been affected by the pandemic by increasing the comorbidity of older people [29]. Among the quality of life scales, the Euro-Qol 5D scale stands out, designed to assess health status [30] and measure health-related quality of life in people [31]. Even though it has been shown that individualized care has benefits for both nursing professionals and older people, there are hardly any studies that analyze this phenomenon and compare the relationship between quality of life and individualized care, especially in the field of long-term care for older people [20].

The COVID-19 pandemic particularly affected institutionalized older people [9]. Therefore, it is necessary to carry out studies in this group that help us understand the impact of the pandemic on the individualized care of older people and the related factors [18].

The aim of this study is to understand the changes in the perceptions of older people about the individualization of care, their quality of life, and the care environment in long-term care centers before and after the COVID-19 pandemic.

## 2. Materials and Methods

### 2.1. Study Design

A cross-sectional prospective observational study was conducted to analyze the perceptions of older people about the individualization of care, their quality of life, and the care environment in public long-term care centers for the elderly. First, the centers were informed about the objectives of the study. Afterwards, voluntary centers were recruited for participation, and formal authorization was subsequently requested from the director of the center. Data collection was conducted in two phases: the first from September 2019 until the declaration of the pandemic in March 2020 (in Spain) and the second from September 2022 to March 2023. No data were collected between phases due to COVID-19 health restrictions that limited visits to the centers.

### 2.2. Participants

An intentional sample of older people institutionalized in public long-term care centers for older people in Castilla La-Mancha (Spain) was used, who agreed to voluntarily participate in the study and met the following inclusion criteria: (1) to be permanently institutionalized in a public long-term care center participating in the study, (2) to have the ability to understand and complete the questionnaires, (3) to agree to participate voluntarily in the study, and (4) to sign the informed consent document. The exclusion criteria were as follows: (1) to have cognitive impairment, (2) to be legally incapacitated, and (3) to suffer from a physical or psychological pathology that would prevent their participation in the study.

### 2.3. Data Collection Instruments

A booklet containing a set of questionnaires was provided to the participants. Each questionnaire was specifically selected to address each research objective. To understand the general characteristics of the participants, their sociodemographic data were collected (age, sex, educational level, and length of residence in the center). To analyze the impact of the pandemic on the perceptions of the subjects interviewed after it, they were asked whether COVID-19 had positively, negatively, or indifferently affected their perceptions.

To evaluate the perceptions of older people about the individualization of care, the ICS-patient was used. This scale consists of two subscales that address different moments in the provision of individualized care: usual practice (Part A) and recent experience (Part B). Each subscale includes 17 items, evaluated through a Likert-type scale that is divided into three main categories to evaluate: the clinical situation, the personal situation, and the degree of control in decision-making. This questionnaire, which is central to this study, is validated in Spanish so that its psychometric properties of reliability and validity are assessed [27].

The evaluation of the quality of life in older people in relation to their current health status was carried out using the Euro-Qol 5D questionnaire. This instrument provides a classification of five dimensions: mobility, personal care, daily activities, pain/discomfort, and anxiety/depression. Each dimension has three levels: no problems, moderate problems, and extreme problems [32]. It is important to note that the five dimensions of quality of life have been evaluated in the general context of the participants’ well-being, without any special implications related to COVID-19. In addition, it provides a self-assessment thermometer of health status.

Finally, the characteristics of the environment and its relationship with the perception of care was analyzed using an abbreviated version of the Sheffield Care Environment Assessment Matrix (A-SCEAM). This questionnaire is composed of five domains that collect relevant characteristics for people cared for in a specific environment. Characteristics are evaluated according to their presence or absence in the environment [33].

### 2.4. Ethical Considerations

This study complied with the Helsinki Declaration and with the current regulations on personal data (Organic Law 3/2018, of 5 December, on Personal Data Protection and guarantee of digital rights) [34]. It was also approved by the Ethics Committee for Research with medications of the Integrated Care Management of Ciudad Real, Spain (Date 23 July 2019, Agreement 07/2019, ID C-269) and by the directors of the participating centers. All participants provided their informed consent in writing after a complete and adapted explanation of the project by the principal investigator.

### 2.5. Statistical Analysis

An exploratory and inferential analysis was performed using the software Statistical Package for Social Sciences v.28 (SPSS 28). The demographic characteristics of the participants, Euro-Qol 5D, A-SCEAM, and ICS-patient scores were first studied through a descriptive analysis. Differences in the frequency distribution between categorical variables were evaluated through Fisher’s exact test, while the median test was the nonparametric test selected for exploring the discrepancies between numerical variables. Fisher’s exact test is the most appropriate technique for analyzing the dependence or independence of qualitative variables compared to others, such as the chi-square test, as it is valid for any sample size. While the chi-squared test relies on approximation, Fisher’s exact test is one of the exact tests [35]. Regarding numerical variables, the ordinal nature of Likert scale data implies that the assumptions of parametric tests, such as normality of distribution or equality of variances, are not always applicable [36]. Thus, the median test is a non-parametric test that provides robust decisions with ordinal data, such as those in this paper [37]. The purpose of this test is to compare whether the medians of two or more independent groups differ significantly. All statistical evaluations were considered significant at level of 0.05.

## 3. Results

### 3.1. Sample Description

A total of 177 elderly individuals residing in long-term care facilities participated in the study, of which 87 completed the questionnaires before the pandemic and 90 after it. Table 1 summarizes the main demographic features of the participants (see Table 1). The average age of participants was 83.3 years old, specifically 82.2 and 84.27 pre- and post-pandemic, respectively. Most of the residents were females (62.7%) and reported not having formal education (62.7%). The average length of residence in the institution (up to the moment of the interview) was 4.5 years. Subjects interviewed after COVID-19 mostly reported not having been affected by the pandemic (54.4%), and only 36.7% of them stated that the pandemic affected them negatively.

### 3.2. Life Quality Pre- and Post-Pandemic

Table 2 gathers the results about the perceived health-related quality of life of the participants. Most of them reported having moderate problems with mobility (60.8%), no problems with self-care (57.7%) and performing their usual activities (61.6%). Nevertheless, nearly two out of three residents stated having moderate pain or discomfort. Regarding the anxiety or depression dimension of the test, 46.9% of participants reported no anxiety or depression, 43.5% reported moderate levels, and 9.6% reported extreme levels. The average self-evaluation was 6.47 (±2.28) points out of 10, being 6.02 and 6.91 pre- and post-pandemic, respectively. The median test revealed no significant differences in the self-evaluation pre- and post-pandemic (*p* = 0.058). Therefore, the health-related quality of life of the participants did not experience substantial changes after the pandemic.

### 3.3. Individualized Care Pre- and Post-Pandemic in the Ordinary Practice (ICS-A)

**Figure 1:**
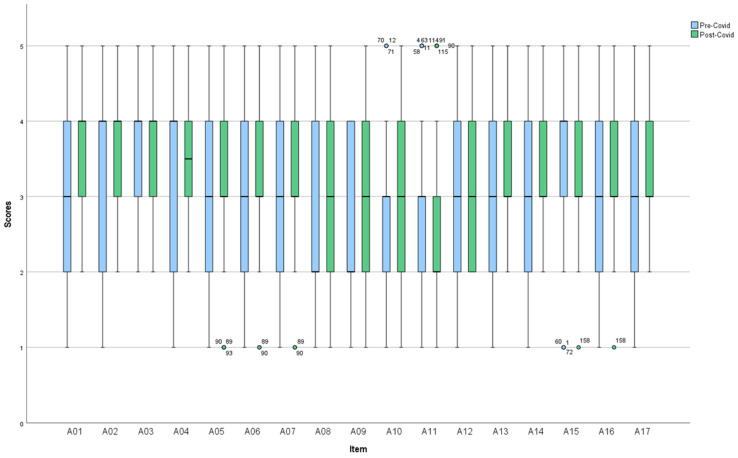
Description of ICS-A-patient items.

Table 3 reports the results of nursing activities during their general activity (ICS-A). Items related to the clinical situation were rated with a median of 4 or 3, expressing moderate or indifferent agreement to the care provided, respectively. In particular, items related to the understanding of the disease and fears were scored lower than those related to care needs and responsibilities. Regarding personal life situation, the median was 3 in all items except for item 11. Most residents expressed disagreement with the degree of family involvement in care. Decisional control was rated indifferently across all items. Figure 1 visually displays the items in which significant differences were obtained in the scores before and after the pandemic. Specifically, these were items A01, A02, and A07, all of which belong to the clinical situation dimension. The first two items assess the expression of feelings and needs for care, while item A07 is related to the meaning of the illness. All of them scored slightly higher post-COVID-19 than pre-COVID-19. Thus, the experience lived during the pandemic has sensitized nursing professionals regarding the clinical situation of their patients, favoring communication with residents about their care.

### 3.4. Individualized Care Pre- and Post-Pandemic in the Recent Experience (ICS-B)

Table 4 reports the results about the maintenance of individuality in the care they provided (e.g., last shift) (ICS-B). It is noteworthy that the median score of all items (except item 11) was 3, exhibiting an indifferent agreement regarding all aspects analyzed of the individualization of care. This result proves that there is still much to improve. Once again, item 11 related to family intervention in care is poorly rated with 2 points, showing a majority disagreement.

**Figure 2:**
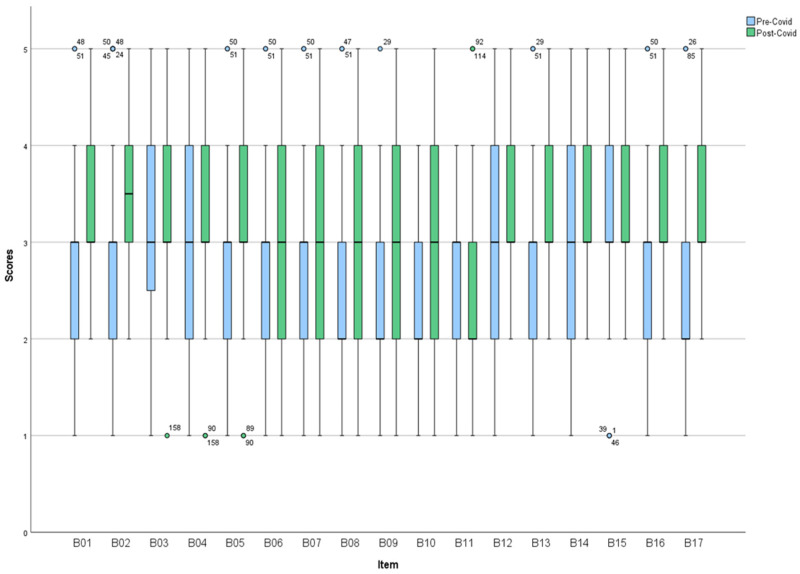
Description of ICS-B-patient items.

Figure 2 reveals significant differences between scores pre- and post-COVID-19 in the items B01, B02, B05, B06, B07, B08, B09, B10, B16, and B17 (see Figure 2). In recent experience, median scores were significantly higher post-COVID-19 not only in certain aspects of the clinical situation, as was the case in usual practice (part A), but also in most items of the personal life situation and other decision-making aspects, such as the expression of opinions. This reinforces the idea that COVID-19 has improved certain aspects of care in recent experiences. Regarding deciding when to bathe (B17), a woman interviewed before the pandemic stated: “I cannot attend mass on Sundays according to my preferences because it is my turn for the bathroom”. This kind of action penalized the scores before the pandemic. On the other hand, care and management of nursing homes were severely criticized in Spain during the pandemic, so perhaps some kind of actions could have been taken after COVID-19. However, this result should be interpreted with caution since the averages oscillate between 2 and 3 points out of 5, indicating that there is still much room for improvement.

### 3.5. Comparing Individualized Care in the Ordinary Practice and the Recent Experience (ICS-A vs. ICS-B)

No noticeable differences were observed between the results obtained in subscales A and B (Table 3 and Table 4, respectively). It can only be highlighted that the median scores of most of the clinical situation items were better rated (4) during the general activities of care (ICS-A subscale) than in the last shift (3) (ICS-B subscale).

### 3.6. Care Enviroment Pre- and Post-Pandemic

Results derived from the perceptions about the care environment before and after the pandemic are collected in Table 5. Most respondents reported having enough privacy (70.6%), being able to customize their spaces according to their preferences (76.3%), having control over their preferences (74%), and having a good relationship with other residents (85.3%). Regarding physical issues, around 90% of those interviewed stated that they felt safe in terms of healthcare and comfortable in the residence place. Moreover, they reported having received support according to their physical conditions. About three out of four residents participated in activities offered by the institution to work on memory, language, thinking, and judgement, and approximately one of three respondents were aware of the temporary situation. In general, a vast majority (94.4%) considered the residence environment suitable. Around 90% of participants considered the cleaning, maintenance, healthcare, and general attention of the residence to be proper, whereas the eating regimen was approved by 72.9% of them. Regarding facilities, it is noteworthy that although 78% of residents have a single room, only 39.5% of them have a single bathroom. Almost all the respondents considered there to be sufficient width in the corridors and enough space for them in the common places (97.7% and 95.5%, respectively). Only one out of three selected institutions was in an urban core, while all of them had outdoor areas. Significant differences were detected in the frequency distributions pre- and post-COVID-19 regarding the relationship with other residents, comfort in the residence place, mental support, cleaning, eating regimen, maintenance, single rooms, spaces in common places, and location in an urban core (marked with *C in Table 5). In general, all these issues improved to a greater or lesser extent after the pandemic except for the availability of single rooms, which was drastically reduced after COVID-19 (from 93.1% to 63.3%).

### 3.7. Individualized Care vs. Life Quality

Relations between average scores obtained in the ICS-A and ICS-B and the different analyzed aspects of patient life quality were examined. Significant differences in ICS-A scores or ICS-B scores were marked with *A or *B in Table 2. Patients without mobility, self-care, usual activity, and anxiety problems scored higher in both the ICS-A and ICS-B than those with moderate problems. In general, the patients unable to perform previous activities were the group that more harshly penalized the scores given to individualized care. No significant differences were found in the ICS averages depending on the level of patient pain nor dimensions A and B of the ICS test. The Spearman correlation coefficient was used to measure the relationship between self-evaluation and ICS scores. It was 0.137 and 0.171 for the ICS-A and ICS-B, respectively. Nevertheless, in the second case, the observed relation turns out to be significant (*p* = 0.023) but not in the first case (*p* = 0.07). It is noteworthy that in both cases, the exhibited relationship is very weak so that it cannot be considered that there is a relationship between the ICS averages and the self-evaluation provided in the Euro-Qol 5D.

### 3.8. Individualized Care vs. Care Enviroment

To explain the influence of the care environment over the individualized care perception, the relations between the groups established by A-SCEAM and the ICS average scores were also analyzed (see Table 5, significant differences were marked with *A or *B). In general, the social environment is highly influential in the perception of care. Patients feeling control over their preferences or privacy perceive better care. The same occurs with those who feel comfortable in the residence place and have a good temporal awareness. Professionals turned out to be highly influential in the individual care perception. In particular, residents considering good cleaning, health, and maintenance professionals in their institutions exhibited a higher average score. Regarding facilities, the fact of having a single bathroom or the location in an urban core also showed relation with a better perception in the individualization of care. It is worth mentioning that differences between scores regarding security in terms of health care were significant only in the ICS-A (habitual care practice) and regarding maintenance in the ICS-B (last shift). Nevertheless, most of the A-SCEAM items exhibited the same behavior in both dimensions of the ICS.

## 4. Discussion

This study provides evidence on the perceptions of institutionalized older people in long-term care centers about the individualization of care, their quality of life, and care environment before and after the COVID-19 pandemic. The results obtained show that perceptions about individualized care before and after the pandemic are similar, except in aspects such as the participation of relatives in care, decreasing the post-pandemic score, or the expression of feelings by the respondent, increasing their scores after the pandemic. In addition, we observe that the higher the quality of life, the better the perception of institutionalized older people about individualized care. Likewise, perceiving the care environment as adequate is associated with a better perception of the individualization of care.

No significant differences were found in the scores on most of the ICS-patient questionnaires before and after the pandemic or between their subscales A and B, although some discordant aspects should be noted. During the pandemic, long-term care centers for the elderly recorded large peaks in mortality, this being attributed in part to the high comorbidity of the residents but also to poor management of the pandemic and a lack of resources [38]. These aspects influence the perception of the participants, as more than 25% of the participants report that the pandemic affected them. In some cultures, for example in Southern European countries, family involvement is deeply rooted in elderly care, so pandemic restrictions had a more pronounced psychological impact on residents and caregivers [38]. This situation has generated modifications in health policies and action plans of institutions, with the purpose of optimizing care [39]. Among the measures implemented in long-term care centers during the pandemic, the measures of isolation of residents in their rooms, the prohibition of visits from relatives and friends, or changes in the care model stand out, to which was added the lack of nursing professionals due to the high number of layoffs [40]. The results of this study reflect these measures, as older people demand that their relatives can be involved in their care again. Family-centered care strategies, such as flexible visitation policies and technology for virtual interaction, which have been linked to better clinical outcomes and more personalized care plans, have been suggested [41]. The COVID-19 pandemic highlighted the negative effects of reduced family interaction. Innovative approaches, like smart cameras or AI-driven tools, are being considered to enable families to remain involved while addressing privacy and autonomy concerns of residents [42]. Since the benefits of new technologies in family interaction have been demonstrated, this study proposes that equipping residential facilities with technological tools could be a good measure to adopt. On the other hand, certain aspects linked to the change in the care model during the pandemic have influenced the improvements perceived by the participants after it, as older people have expressed their feelings and have requested care when required in a more open way to nursing professionals, although the scores have been low with respect to the other items. The lack of attention to the personal life of older people by nursing professionals may be influenced by several factors related to the challenges faced by professionals. One important reason could be the high workload due to a lack of staff, stress level, insufficient training, and a lack of resources experienced by nursing staff. Efforts to address these gaps have highlighted the importance of health promotion programs that not only focus on the well-being of patients but also on supporting nurses themselves through better working conditions and resources for their physical and mental health [43]. Following the line of other studies carried out on quality of life [38], our results confirm that the higher the quality of life, the greater the perception of the individualization of care. In addition, this study provides evidence that the fewer daily life activities the participants can perform, the worse their perception of the individualization of care.

We agree with a previous study conducted during the pandemic [40] on the importance of conducting an individualized assessment of institutionalized older people in long-term care centers that includes their needs and preferences as well as the need to incorporate the family and nursing professionals as relevant protagonists of care, justified by the desire of the participants to involve their relatives in their care. Despite the end of the state of alarm in May 2021, which allowed visits to long-term care centers in Spain again [44], most centers continue to maintain pandemic restrictions, so in many center visits are not allowed, and in others, there is a set schedule for them, prior authorization is needed, or the place of the visit is a specific area of the center enabled for it [45]. Strict visitation bans implemented in many countries, except the UK, had mixed impacts. While effective in controlling infection, they negatively affected residents’ mental health. Countries such as Denmark managed to quickly return to pre-pandemic care models, while others are still struggling to balance infection control and individualized care [46]. These rules negatively influence the participants’ perception related to the participation of their relatives in their care, as they do not have the same possibility as before the pandemic to be involved and participate in care. The results, which follow the line of other studies, show the low level of patient satisfaction due to social distancing [47], pointing to the need to develop innovative interventions to improve social connections [48].

As a result of this study, one of the actions to improve the perception of individualized care could be the implementation of interventions aimed at better understanding the resources and facilities available to long-term care centers. In this sense, although most of the items on the Sheffield Care Environment Assessment Matrix questionnaire improve after the pandemic, there are some aspects to highlight (individual rooms, individual bathrooms…). A previous study states that due to the isolation imposed during the first phase of the pandemic, individual rooms were prioritized in long-term care centers [49]. This finding was not found in our study, as in most cases, residents did not have an individual bathroom, and after the pandemic, paradoxically, the number of individual rooms in the centers has decreased. This may have been due to the restructuring of common areas in care homes during the pandemic to ensure the safety and well-being of residents. The remodeling of spaces may have resulted in the elimination of individual rooms or bathrooms. Research indicates that the measures implemented included reorganizing spaces to maintain social distancing, reducing the number of individuals in shared areas, and designing protocols to minimize close contact, such as staggered schedules for meals and activities [50,51]. Various studies link overcrowding with a higher probability of experiencing larger and more deadly COVID-19 outbreaks [49,52]. Therefore, to avoid possible respiratory outbreaks, it is necessary for centers to have more individual rooms and bathrooms. To do this, it is necessary to contribute to the individualization of care by nursing staff [53] and thus promote the privacy of the resident [54].

It is known that the environment is a fundamental factor for improving the quality of life and care [24]. As previously pointed out, our results confirm that older people who have control over their preferences and privacy and who are satisfied with the environment and services provided coincide in perceiving better individualization of care [54]. All participating centers have places to be outdoors, but in most cases, they are not located in the urban center. We know that the community integration of long-term care centers is fundamental to avoid the isolation of residents and promote their social interaction, and being able to be closer to friends and family also increases their quality of life [29]. In addition, the accessibility and availability of resources for daily activities as well as medical assistance contribute to improving the autonomy of the elderly person [55] and increasing their quality of life, which, in turn, favors the individualization of care according to the results of our study.

Recent studies have explored the clinical implications of individualized care for older adults in residential settings. Research highlights that in older people, specifically those with dementia, clinical outcomes are improved, and behavioral symptoms are reduced by improving factors such as the organizational environment, staff training, and individualized care planning, such as in Australia’s Harmony in the Bush initiative [56]. Another review identifies promising practices to address social isolation and loneliness, common problems exacerbated during the pandemic. Interventions such as digital communication tools, structured social activities, and family inclusion are recognized as vital to improving residents’ psychological and physical health in the wake of the pandemic [57]. In this regard, this study suggests establishing clear contingency plans as well as conducting regular drills and training sessions to prepare nursing staff for potential crises. Continuous training in technological tools should be a priority, along with the implementation of effective communication and socialization strategies to ensure humanized care.

One of the limitations of this study is that it was not possible to access the sample in private long-term care centers due to the severe entry restrictions after the COVID-19 pandemic. The highest mortality rate during the pandemic in Spain were recorded in this type of center [58]. Studies comparing individualized care in private and public nursing homes reveal key differences that can significantly affect the quality of care. Public nursing homes typically receive more government funding, which can lead to higher staffing ratios and greater quality control. In contrast, private nursing homes, while often better equipped and offering more personalized services, tend to be more expensive and less accessible to people without adequate financial resources [59]. Another limitation is that only fully competent participants (in physical and psychological faculties) were included in the study so that it could affect the type of care they required. Future studies should include highly dependent people in order to make comparisons. It is also important to consider that due to the sudden and unpredictable nature of the COVID-19 situation combined with the confinement measures, patient recruitment in the pre-pandemic phase was abruptly halted, resulting in a relatively small sample size. This limitation should be considered when interpreting the scope of this study. On the other hand, the analyzed sample has a clear female predominance, which reflects the feminization of old age in our country, especially in the most advanced age ranges.

## 5. Conclusions

The results of this study reflect no significant changes in the perception of individualized care in older people before and after the COVID-19 pandemic. Nevertheless, scores were low or moderate in both temporal moments so that there is room for improvement in the management system. Factors such as interpersonal communication between nursing staff and the elderly person were slightly improved after the pandemic, but there are others such as family intervention or the availability of more individual spaces in the residences that need to be reinforced.

## Figures and Tables

**Table 1:** The description of sociodemographic variables of the sample.

	Pre-COVID-19	Post-COVID-19	*p*	All
**N**	87 (49.2%)	90 (50.8%)		177 (100%)
**Age Mean (SD)**	82.2 (±12.3)	84.27 (±7.2)	0.598	83.3 (±10.1)
**Gender**			0.084	
Male	38 (43.7%)	28 (31.1%)		66 (37.3%)
Female	49 (56.3%)	62 (68.9%)		111 (62.7%)
**Residence time (in ages)**	4.6 (±3.9)	4.4 (±4.5)	0.335	4.5 (± 4.2)
**Education**			0.419	
No education	51 (58.6%)	60 (66.7%)		111 (62.7%)
Primary studies	32 (36.8%)	29 (32.2%)		61 (34.5%)
University studies	4 (4.5%)	1 (1.1%)		5 (2.9%)
**COVID-19 pandemic affectation**				
Positively		8 (8.9%)		
Negatively		33 (36.7%%)		
No affectation		49 (54.4%)		

Abbreviations: *p*: *p*-value resulting from the median test and Fisher’s exact test for numerical and categorical variables, respectively.

**Table 2:** Life quality through EuroQol-5D.

	Pre-COVID-19	Post-COVID-19	All	$\bar{x}$ ICS-A	*p* ICS-A	$\bar{x}$ ICS-B	*p* ICS-B
Mobility *A, *B					<0.001		0.003
No problems in walking about	24 (27.9%)	40 (44.4%)	64 (36.4%)	3.43		3.19	
Moderate problems in walking about	58 (67.4%)	49 (54.4%)	107 (60.8%)	3.01		2.85	
Unable to walk about	4 (4.7%)	1 (1.1%)	5 (2.8%)	2.03		2.05	
Self-care *A, *B					<0.001		<0.001
No problems with washing or dressing myself	41 (47.1%)	61 (67.8%)	102 (57.7%)	3.34		3.13	
Moderate problems with washing or dressing myself	34 (39.1%)	27 (30%)	61 (34.5%)	3		2.84	
Unable to wash or dress myself	12 (13.8%)	2 (2.2%)	14 (7.9%)	2.21		2.08	
Usual activities *A, *B					0.008		0.006
No problems doing my usual activities	45 (51.7%)	64 (71.1%)	109 (61.6%)	3.23		3.06	
Moderate problems doing my usual activities	34 (39.1%)	26 (28.9%)	60 (33.9%)	3.10		2.88	
Unable to do my usual activities	8 (9.2%)	0 (0%)	8 (4.5%)	2.29		2.21	
**Pain/Discomfort**					0.850		0.828
No pain or discomfort	22 (25.3%)	25 (27.8%)	47 (26.6%)	3.13		2.89	
Moderate pain or discomfort	54 (62.1%)	59 (65.6%)	113 (63.8%)	3.17		3.01	
Extreme pain or discomfort	11 (12.6%)	6 (6.7%)	17 (9.6%)	2.99		2.81	
Anxiety/Depression *A, *B					0.001		0.001
No anxiety or depression	31 (35.6%)	52 (57.8%)	83 (46.9%)	3.41		3.20	
Moderate anxiety or depression	40 (46.0%)	37 (41.1%)	77 (43.5%)	2.88		2.73	
Extreme anxiety or depression	16 (18.4%)	1 (1.1%)	17 (9.6%)	3.08		2.74	
**Self-evaluation**	6.02 (±2.42)	6.91 (±2.06)	6.47 (±2.28)				

Abbreviations: ICS-A: Individualized Care Scale-patient Scale A; ICS-B: Individualized Care Scale-patient Scale B; *p*: *p*-value resulting from median test. *A and *B indicate significant differences in the averages of the ICS-A or ICS-B scores, respectively, for each dimension of EuroQol-5D.

**Table 3:** Description of ICS-A-patient items.

	ICS-A-Pre-COVID-19			ICS-A-Post-COVID-19				ICS-A-All		
	Mean (±SD)	Median	Range	Mean (±SD)	Median	Range	*p*	Mean (±SD)	Median	Range
**Clinical situation**	3.12 (±0.72)	3	1–5	3.51 (±0.47)	4	1–5	0.408	3.32 (±0.59)	4	1–5
1. Feelings *	3.09 (±1.11)	3	1–5	3.58 (±0.95)	4	2–5	0.003	3.34 (±1.05)	4	1–5
2. Needs for care *	3.20 (±1.14)	4	1–5	3.60 (±1.02)	4	2–5	0.039	3.40 (±1.09)	4	1–5
3. Responsibility for care	3.66 (±1.06)	4	2–5	3.66 (±0.95)	4	2–5	0.948	3.66 (±1.00)	4	2–5
4. Changes in conditions	3.36 (±1.17)	4	1–5	3.56 (±0.95)	3.5	2–5	0.618	3.46 (±1.07)	4	1–5
5. Fears and anxieties	2.76 (±1.03)	3	1–5	3.41 (±1.06)	3	1–5	0.057	3.09 (±1.09)	3	1–5
6. Effects of the illness	2.93 (±1.10)	3	1–5	3.40 (±1.06)	3	1–5	0.137	3.17 (±1.10)	3	1–5
7. Meaning of the illness *	2.87 (±1.02)	3	1–5	3.40 (±1.09)	3	1–5	0.035	3.14 (±1.09)	3	1–5
**Personal life situation**	2.60 (±0.84)	2.5	1–5	2.86 (±0.60)	3	1–5	0.770	2.74 (±0.72)	2.5	1–5
8. Activities in daily life	2.72 (±1.18)	2	1–5	2.97 (±1.07)	3	1–5	0.634	2.85 (±1.13)	3	1–5
9. Hospital experience	2.45 (±1.13)	2	1–4	2.96 (±0.90)	3	1–5	0.892	2.71 (±1.05)	3	1–5
10. Everyday habits	2.64 (±1.03)	3	1–5	3.08 (±1.01)	3	1–5	0.220	2.86 (±1.04)	3	1–5
11. Family in care	2.60 (±1.16)	3	1–5	2.46 (±0.82)	2	1–5	0.086	2.53 (±1.00)	2	1–5
**Decisional control**	3.07 (±0.69)	3	1–5	3.35 (±0.50)	3	1–5	0.590	3.21 (±0.59)	3	1–5
12. Information	3.03 (±1.16)	3	1–5	3.37 (±1.04)	3	2–5	0.683	3.20 (±1.11)	3	1–5
13. Want to know	3.05 (±1.07)	3	1–5	3.37 (±0.97)	3	2–5	0.134	3.21 (±1.03)	3	1–5
14. Personal wishes	3.29 (±1.32)	3	1–5	3.39 (±1.01)	3	2–5	0.804	3.34 (±1.17)	3	1–5
15. Decision making	3.31 (±0.98)	4	1–5	3.31 (±0.99)	3	1–5	0.415	3.31 (±0.98)	3	1–5
16. Express opinion	2.98 (±1.05)	3	1–5	3.31 (±1.00)	3	2–5	0.907	3.15 (±1.02)	3	1–5
17. Want to wash	2.78 (±1.20)	3	1–5	3.37 (±0.83)	3	2–5	0.065	3.08 (±1.09)	3	1–5
**Total**	2.98 (±0.72)	3	1–5	3.30 (± 0.73)	3	1–5	0.598	3.14 (±0.74)	3	1–5

Abbreviations: ICS-A: Individualized Care Scale-patient Scale A; SD: Standard Deviation. *p*: *p*-value resulting from median test; *: Significant differences at 0.05 level. © This instrument has copyright reserved. Riitta Suhonen 2007.

**Table 4:** Description of ICS-B-patient items.

	ICS-B-Pre-COVID-19			ICS-B-Post-COVID-19				ICS-B-All		
	Mean (±SD)	Median	Range	Mean (±SD)	Median	Range	*p*	Mean (±SD)	Median	Range
Clinical situation *	2.76 (±0.54)	3	1–5	3.34 (±0.42)	3	1–5	<0.001	3.06 (±0.48)	3	1–5
1. Feelings *	2.69 (±0.91)	3	1–5	3.36 (±0.92)	3	2–5	<0.001	3.03 (±0.97)	3	1–5
2. Needs for care *	2.72 (±1.08)	3	1–5	3.46 (±0.99)	3.5	2–5	<0.001	3.10 (±1.09)	3	1–5
3. Responsibility for care	3.22 (±0.98)	3	1–5	3.43 (±0.90)	3	1–5	0.920	3.33 (±0.94)	3	1–5
4. Changes in conditions	2.84 (±1.09)	3	1–5	3.39 (±0.97)	3	1–5	0.065	3.12 (±1.06)	3	1–5
5. Fears and anxieties *	2.72 (±0.97)	3	1–5	3.29 (±1.00)	3	1–5	0.029	3.01 (±1.02)	3	1–5
6. Effects of the illness *	2.55 (±0.93)	3	1–5	3.22 (±1.03)	3	1–5	<0.001	2.89 (±1.03)	3	1–5
7. Meaning of the illness *										
Personal life situation *	2.48 (±0.65)	2.5	1–5	2.87 (±0.52)	3	1–5	0.026	2.68 (±0.59)	2.5	1–5
8. Activities in daily life *	2.45 (±0.94)	2	1–5	2.90 (±1.01)	3	1–5	0.018	2.68 (±1.00)	3	1–5
9. Hospital experience *	2.34 (±0.90)	2	1–5	2.99 (±0.91)	3	1–5	0.002	2.67 (±0.96)	3	1–5
10. Everyday habits *	2.51 (±0.90)	2	1–4	3.02 (±0.94)	3	1–5	0.044	2.77 (±0.95)	3	1–5
11. Family in care	2.63 (±86)	3	1–4	2.56 (±0.81)	2	1–5	0.261	2.59 (±0.84)	2	1–5
Decisional control *	2.80 (±0.71)	3	1–5	3.25 (±0.39)	3	2–5	<0.001	3.03 (±0.55)	3	1–5
12. Information	2.86 (±0.99)	3	1–5	3.33 (±1.01)	3	2–5	0.216	3.10 (±1.02)	3	1–5
13. Want to know	2.80 (±0.93)	3	1–5	3.20 (±0.89)	3	2–5	0.095	3.01 (±0.93)	3	1–5
14. Personal wishes	2.97 (±0.98)	3	1–5	3.23 (±0.89)	3	2–5	0.342	3.10 (±0.94)	3	1–5
15. Decision making	3.34 (±0.95)	3	1–5	3.26 (±0.89)	3	2–5	0.338	3.30 (±0.92)	3	1–5
16. Express opinion *	2.62 (±0.89)	3	1–5	3.19 (±0.87)	3	2–5	0.004	2.91 (±0.93)	3	1–5
17. Want to wash *	2.21 (±0.88)	2	1–5	3.29 (±0.92)	3	2–5	<0.001	2.76 (±1.05)	3	1–5
**Total**	2.71 (±0.63)	2	1–5	3.20 (±0.71)	3	1–5	<0.001	2.96 (±0.72)	3	1–5

Abbreviations: ICS-B: Individualized Care Scale-patient Scale B; SD: Standard Deviation; *p*: *p*-value resulting from median test; *: Significant differences at 0.05 level. © This instrument has copyright reserved. Riitta Suhonen 2000.

**Table 5:** Abbreviated Sheffield Care Environment Assessment Matrix (A-SCEAM).

	Pre-COVID-19	Post-COVID-19	*p* pre/post	All	$\bar{x}$ ICS-A	*p* ICS-A	$\bar{x}$ ICS-B	*p* ICS-B
**A. Social**								
Enough privacy? *A, *B			0.132			<0.001		<0.001
Yes	66 (74.9%)	59 (65.6%)		125 (70.6%)	3.25		3.08	
No	21 (24.1)	34.4%		52 (29.4%)	2.89		2.67	
Customized to your preferences? *A, *B			0.561			0.009		0.001
Yes	68 (78.2%)	67 (74.4%)		135 (76.3%)	3.25		3.08	
No	19 (21.8%)	23 (25.6%)		42 (23.7%)	2.77		2.56	
Control over your preferences? *A, *B			0.216			<0.001		<0.001
Yes	68 (78.2%)	63 (70.0%)		131 (74%)	3.30		3.12	
No	19 (21.8%)	27 (30.0%)		46 (26%)	2.70		2.49	
Good relationship with other residents? *C			0.027			0.303		0.165
Yes	69 (79.3%)	82 (91.1%)		151 (85.3%)	3.20		3.00	
No	18 (20.7%)	8 (8.9%)		26 (14.7%)	2.83		2.66	
**B. Physical**								
Feel safe in terms of health care? *A			0.869			0.04		0.308
Yes	78 (89.7%)	80 (88.9%)		158 (89.3%)	3.21		3.00	
No	9 (10.3%)	10 (11.1%)		19 (10.7%)	2.60		2.62	
Support according to your physical conditions?			0.229			0.627		0.103
Yes	81 (93.1%)	79 (87.8%)		160 (90.4%)	3.15		2.98	
No	6 (6.9%)	11 (12.2%)		17 (9.6%)	3.11		2.78	
Feel comfortable in your residence place? *C, *A, *B			<0.001			0.044		0.032
Yes	71 (81.6%)	89 (98.9%)		160 (90.4%)	3.22		3.03	
No	16 (18.4%)	1 (1.1%)		17 (9.6%)	2.42		2.30	
**C. Mental**								
Support to work memory, language, thinking and judgement? *C			0.028			0.829		0.657
Yes	58 (66.7%)	73 (81.1%)		131 (74.0%)	3.16		3.00	
No	29 (33.3%)	17 (18.9%)		46 (26.0%)	3.10		2.84	
Temporal awareness? *A, *B			0.077			<0.001		<0.001
Yes	54 (62.1%)	67 (74.4%)		121 (68.4%)	3.33		3.14	
No	33 (37.9%)	23 (25.6%)		56 (31.6%)	2.74		2.55	
Suitable residence environment?			0.480			0.338		0.990
Yes	81 (93.1%)	86 (95.6%)		167 (94.4%)	3.16		2.98	
No	6 (6.9%)	4 (4.4%)		10 (5.6%)	2.78		2.61	
**D. Professionals**								
Proper cleaning? *C, *A, *B			0.045			0.004		0.048
Yes	79 (90.8%)	88 (97.8%)		167 (94.4%)	3.19		3.00	
No	8 (9.2%)	2 (2.2%)		10 (5.6%)	2.38		2.31	
Proper eating regimen? *C			0.030			0.140		0.456
Yes	57 (65.5%)	72 (80.0%)		129 (72.9%)	3.21		3.02	
No	30 (34.5%)	18 (20.0%)		48 (27.1%)	2.96		2.78	
Proper maintenance? *C, *B			0.015			0.591		0.014
Yes	72 (82.8%)	86 (95.5%)		158 (89.3%)	3.19		3.01	
No	15 (17.2%)	4 (4.5%)		19 (10.7%)	2.77		2.56	
Proper healthcare? *A, *B			0.358			<0.001		0.021
Yes	80 (92.0%)	79 (87.8%)		159 (89.8%)	3.24		3.04	
No	7 (8.0%)	11 (12.2%)		18 (10.2%)	2.25		2.22	
Proper attention? *A, *B			0.515			<0.001		0.013
Yes	79 (90.8%)	79 (87.8%)		158 (89.3%)	3.27		3.06	
No	8 (9.2%)	11 (12.2%)		19 (10.7%)	2.11		2.13	
**E. Facilities**								
Single room? *C			<0.001			0.968		0.63
Yes	81 (93.1%)	57 (63.3%)		138 (78.0%)	3.16		2.95	
No	6 (6.9%)	33 (36.7%)		39 (22.0%)	3.08		2.97	
Single bathroom? *A, *B			0.624			0.003		0.006
Yes	36 (41.4%)	34 (37.8%)		70 (39.5%)	3.38		3.18	
No	51 (58.6%)	56 (62.2%)		107 (60.5%)	2.98		2.81	
Sufficient width of corridors?			0.296			0.132		0.754
Yes	84 (96.6%)	89 (98.9%)		173 (97.7%)	3.14		2.96	
No	3 (3.4%)	1 (1.1%)		4 (2.3%)	3.09		2.79	
Sufficient space for you in common places? *C			0.026			0.730		0.933
Yes	80 (92.0%)	89 (98.9%)		169 (95.5%)	3.16		2.96	
No	7 (8.0%)	1 (1.1%)		8 (4.5%)	2.78		2.87	
Have outdoor areas?			0.324			0.955		0.923
Yes	87 (100%)	100 (100%)		177 (100%)	3.14		2.96	
No	0 (0%)	0 (0%)		0 (0%)	-		-	
Location in an urban core? *C, *A, *B			<0.001			<0.001		<0.001
Yes	87 (100%)	64 (71.1%)		64 (36.2%)	3.61		3.48	
No	0 (0%)	26 (28.9%)		113 (63.8%)	2.88		2.65	

Abbreviations: ICS-A: Individualized Care Scale-patient Scale A; ICS-B: Individualized Care Scale-patient Scale B; *p*: *p*-value resulting from Fisher’s exact test for *p* pre/post and median test for *p* ICS-A and *p* ICS-B. *A and *B indicate significant differences in the averages of the ICS-A or ICS-B scores, respectively, for each dimension of A-SCEAM. *C indicates significant differences in the frequency distributions pre- and post-COVID.

## Data Availability

Dataset available on request from the authors.

## Public Involvement Statement

No public involvement in any aspect of this research.

## Guidelines and Standards Statement

EQUATOR guidelines were followed for reporting observational research by using the STROBE checklist.
